# Reirradiation in progressive high-grade gliomas: outcome, role of concurrent chemotherapy, prognostic factors and validation of a new prognostic score with an independent patient cohort

**DOI:** 10.1186/1748-717X-8-161

**Published:** 2013-07-03

**Authors:** Felix Scholtyssek, Isabella Zwiener, Annika Schlamann, Clemens Seidel, Jürgen Meixensberger, Manfred Bauer, Karl-Titus Hoffmann, Stephanie E Combs, André O von Bueren, Rolf-Dieter Kortmann, Klaus Müller

**Affiliations:** 1 Department of Radiation Oncology, University Medical Center Leipzig, Leipzig, Germany; 2 Institute for Medical Biostatistics, Epidemiology and Informatics, University Medical Center Mainz, Mainz, Germany; 3 Department of Neurosurgery, University Medical Center Leipzig, Leipzig, Germany; 4 Department of Neuropathology, University Medical Center Leipzig, Leipzig, Germany; 5 Department of Neuroradiology, University Medical Center Leipzig, Leipzig, Germany; 6 Department of Radiation Oncology, University Medical Center Heidelberg, Heidelberg, Germany; 7 Department of Pediatrics and Adolescent Medicine, Division of Pediatric Hematology and Oncology, University Medical Center Goettingen, Goettingen, Germany

## Abstract

**Purposes:**

First, to evaluate outcome, the benefit of concurrent chemotherapy and prognostic factors in a cohort of sixty-four high-grade glioma patients who underwent a second course of radiation therapy at progression. Second, to validate a new prognostic score for overall survival after reirradiation of progressive gliomas with an independent patient cohort.

**Patients and methods:**

All patients underwent fractionated reirradiation with a median physical dose of 36 Gy. Median planned target volume was 110.4 ml. Thirty-six patients received concurrent chemotherapy consisting in 24/36 cases (67%) of carboplatin and etoposide and in 12/36 cases (33%) of temozolomide. We used the Kaplan Meier method, log rank test and proportional hazards regression analysis for statistical assessment.

**Results:**

Median overall survival from the start of reirradiation was 7.7 ± 0.7 months. Overall survival rates at 6 and 12 months were 60 ± 6% and 24 ± 6%, respectively. Despite relatively large target volumes we did not observe any major acute toxicity. Concurrent chemotherapy did not appear to improve outcome. In contrast, female gender, young age, WHO grade III histology, favorable Karnofsky performance score and complete resection of the tumor prior to reirradiation were identified as positive prognostic factors for overall survival. We finally validated a recent suggestion for a prognostic score with our independent but small patient cohort. Our preliminary findings suggest that its ability to discriminate between different prognostic groups is limited.

**Conclusions:**

Outcome of our patients was comparable to previous studies. Even in case of large target volumes reirradiation seems to be feasible without observing major toxicity. The benefit of concurrent chemotherapy is still elusive. A reassessment of the prognostic score, tested in this study, using a larger patient cohort is needed.

## Background

Salvage treatment of progressive high-grade gliomas (HGG) remains one of the most challenging tasks in neuro-oncology [1,2]. Meanwhile, reirradiation has been widely accepted as useful therapeutic option and adopted in clinical routine [3]. The credit for this belongs to numerous retrospective studies, which reported satisfactory survival rates and acceptable toxicity in the last two decades. However, most of these studies included highly selected patients focussing on stereotactic reirradiation techniques and the treatment of small tumor volumes. There is only sparse data on reirradiation of large recurrent tumors (Additional file 1: Table S1) [4-10]. Moreover, the benefit of combining reirradiation with concurrent chemotherapy is still elusive [6-8,11] (Additional file 2: Table S2). Assuming significant benefit, what would be the best choice for the chemotherapeutic agent? Temozolomide, a drug which is administered to the vast majority of patients in first-line treatment or an alternative chemotherapy regimen? Our dataset is suitable to contribute to the answers to these important questions. In a recent review, the question which patients would be the best candidates for reirradiation was addressed [12]. This question should be subdivided into two aspects: First, in which patients reirradiation is feasible with acceptable toxicity and second, which patients benefit most from reirradiation in terms of survival? To answer the second question two requirements have to be met:

1. A cohort of reirradiated patients has to be compared with a non-irradiated control group. In this context, we eagerly await the results of the Radiation Therapy Oncology Group (RTOG) 1205 randomized phase II trial (concurrent bevacizumab and reirradiation versus bevacizumab alone as treatment for recurrent glioblastoma).

2. Given the confusing variety of factors determining the outcome of progressive high-grade glioma patients, a simple and reliable system is needed to classify patients into subgroups with similar prognosis. In future these subgroups may constitute the basis for individual patient councelling and clinical decision making (e.g., reirradiation, yes or no?). A German research group recently suggested such a classification system in the form of a prognostic score based on three clinical factors predicting overall survival after reirradiation. However, although perfectly meeting the criterion of simplicity its reliability still had to be validated with an independent patient cohort [3]. In the present analysis we specifically address outcome and toxicity in progressive high-grade glioma patients with large tumor volumes, the role of concurrent chemotherapy, prognostic factors for overall survival and the validation of a simple prognostic score with an independent dataset.

## Patients and methods

Sixty-four patients with recurrent high-grade gliomas underwent reirradiation at our department from March 2005 to May 2012. Gender distribution was almost balanced. Median age at the start of reirradiation was 53.4 years (range: 21.8 – 81.1 years). Median time from the end of the first radiotherapeutic treatment to the start of reirradiation was 13.4 months (range: 2.7 – 202.6 months) (Table 1).

**Table 1:** Characteristics of 64 patients treated for recurrent high-grade gliomas using a second series of radiotherapy with or without concurrent chemotherapy

**Characteristic**	
Median age at Re-RT (years)	53.5
Range: age at Re-RT (years)	21.8 – 81.1
< 50 years at Re-RT	26/64 (40.6%)
≥ 50 years at Re-RT	38/64 (59.4%)
Male	34/64 (53.1%)
Female	30/64 (46.9%)
WHO IV	53/64 (82.8%)
WHO III	11/64 (17.2%)
Karnofsky performance score < 70	13/64 (20.3%)
Karnofsky performance score ≥ 70	51/64 (79.7%)
Median time (RT to Re-RT) (months)	13.4
Range: time (RT to Re-RT) (months)	2.7 – 202.6
Time from RT to Re-RT ≤ 12 months	21/64 (32.8%)
Time from RT to Re-RT > 12 months	43/64 (67.2%)
Concurrent chemotherapy	36/64 (56.3%)
Carboplatin/etoposide	24/64 (37.5%)
Temozolomide	12/64 (18.8%)
Complete resection before Re-RT	8/64 (12.5%)
Incomplete/no resection before Re-RT	56/64 (87.5%)

### First-line treatment

Sixty-three patients (98.4%) underwent neurosurgical resection. All patients received radiotherapy with a median total dose of 60 Gy (range: 36–60 Gy). 56/64 patients (87.5%) received chemotherapy. In 47/56 cases (83.9%) it consisted of temozolomide, which was combined in 2/47 cases (4.3%) with cilengitide. 7/64 patients (10.9%) received second-line chemotherapy and 2/64 patients (3.1%) carmustine implants (gliadel wafers®) during initial treatment.

### Second-line treatment

Fourty-three patients (67.2%) underwent surgical resection or biopsy of the relapsed tumor before reirradiation. In five of those patients even more than one surgery was performed. In 8/64 cases (12.5%) a complete resection was achieved. All patients underwent reirradiation with a median total dose of 36 Gy (range: 30.0 Gy - 40.05 Gy). Fraction doses ranged from 2.0 to 5.0 Gy. Generally, outpatients received 5, hospitalized patients 6 fractions a week. In 58/64 patients (90.6%) conventional 3D-conformal radiotherapy was performed. In contrast, 6/64 patients (9.4%) were treated with stereotactic 3D-conformal radiotherapy.

Gross tumor volume (GTV) was the postsurgical cavity or the macroscopic tumor as seen on pre-/postoperative MRI (enhanced T1 and/or FLAIR/T2). For conventional radiotherapy planning clinical target volume (CTV) was defined as GTV plus a margin of 5 mm and planned target volume (PTV) as CTV plus a margin of 3 – 5 mm.

Median physical total dose of reirradiation was 36.0 Gy (range, 30.0 – 40.05 Gy). Doses per fraction ranged from 2.0 – 5.0 Gy. We used the linear quadratic model to calculate the biologically effective dose (BED) and the normalized total dose (NTD) of each radiation regimen. The BED was calculated according to the following relationship: BED = nd (1 + d/[α/β]) [Gy], with d = fraction dose [Gy], n = number of fractions, nd = D = total physical dose [Gy], and α/β = tissue repair capacity [Gy]. For tumor effects an α/β value of 10 Gy and for normal brain tissue an α/β value of 3 Gy were assumed. In a recent estimation of radiobiologic parameters for malignant gliomas a median α/β value of 9.32 Gy was reported [13]. The BED values were converted to normalized total dose (NTD) values, with NTD being defined as the total dose delivered in 2.0-Gy fractions. The NTD is the ratio of the BED and the relative effectiveness (RE) value. RE = (1 + d/[α/β]), d = 2 Gy [14-16]. The median NTD_(α/β = 10)_ was 39.0 Gy (range, 32.5 – 42.29 Gy). The median NTD_(α/β = 3)_ was 43.2 Gy (range, 36.0 – 48.0 Gy).

Median PTV size was 110.4 ml (range 1.8 ml – 378 ml). In 36 cases chemotherapy was given simultaneously to reirradiation. In 12/36 cases (33%) it consisted of temozolomide (75 mg/m^2^/day) and in 24/36 cases (67%) of carboplatin (100 mg/m^2^/day) and etoposide (120 mg/m^2^/day) (Table 2). In 28/36 patients chemotherapy was continued after reirradiation. 9/64 patients started with chemotherapy after the end of reirradiation (7 with temozolomide and 2 with carboplatin/etoposide). Twelve patients additionally received another chemotherapy regimen and six patients again underwent surgery after reirradation.

**Table 2:** Outcome of 64 patients treated for recurrent high-grade gliomas using a second series of radiotherapy with or without concurrent chemotherapy

**Characteristic**	**OS-6 (%)**	**mOS (months)**	**p=**
< 50 years at Re-RT	73 ± 9	9.4 ± 1.4	0.015
≥ 50 years at Re-RT	50 ± 8	5.8 ± 1.4	
Male	60 ± 9	7.7 ± 0.8	0.669
Female	59 ± 9	7.7 ± 1.7	
WHO IV	55 ± 7	7.4 ± 1.4	0.009
WHO III	82 ± 12	11.2 ± 10.5	
Karnofsky performance score < 70	34 ± 14	5.0 ± 1.0	0.002
Karnofsky performance score ≥ 70	66 ± 7	8.1 ± 0.8	
Time from RT to Re-RT ≤ 12 months	52 ± 11	6.9 ± 2.5	0.140
Time from RT to Re-RT > 12 months	63 ± 8	8.1 ± 1.1	
No concurrent chemotherapy^1^	52 ± 10	6.6 ± 2.9	0.001 (^3^ vs ^2^)
Carboplatin/etoposide^2^	54 ± 10	6.7 ± 1.3	0.006 (^3^ vs ^1^)
Temozolomide^3^	92 ± 8	27.0 ± 17.6	0.455 (^2^ vs ^1^)
Complete resection before Re-RT	100	17.5 ± 3.4	0.034
Incomplete/no resection before Re-RT	55 ± 7	7.4 ± 1.3	

*OS-6* overall survival rate at six months, *mOS* median overall survival (months).

### Histology at primary diagnosis

In one patient the initial histological diagnosis was not available. Besides that, WHO grade III gliomas were diagnosed in 14/64 patients (21.9%) and WHO grade IV glioblastomas/gliosarcomas in 49/64 patients (76.6%).

### Histology at reirradiation

Second surgery and histological assessment revealed WHO grade III gliomas in 4/43 patients (9.3%) and WHO grade IV gliomas in 39/43 patients (90.7%).

### Statistics

Survival times were calculated from the start of reirradiation. For progression-free survival (PFS), events were defined as radiographic or clinical evidence for progression or death by any cause, whatever happened first. Death resulting from any cause was defined as event for overall survival (OS). The latest histological grading (according to the WHO classification of brain tumors, 2007) was used for uni- and multivariate analyses. The Kaplan-Meier method was used to estimate OS and PFS.

### Univariate analyses

Kaplan Meier survival estimates were compared by means of the log rank test. Differences were defined if the p-value was less than 0.05. Aditionally, the influence of continous variables (size of the planned target volume, age and Karnofsky performance score at reirradiation) on OS was assessed using cox proportional hazards regression analysis.

### Multivariate analysis

Cox regression models with backward stepwise selection (inclusion criterion: p-value of the score test ≤ 0.05, exclusion criterion: p-value of the likelihood ratio test ≥ 0.10) were used to analyse the prognostic value of age at the start of reirradiation, Karnofsky performance score (both continuous), sex, extent of prior resection, WHO grading and concurrent chemotherapy on OS [17]. For the final models, the estimated hazard ratios of the selected explanatory variables with respective 95% confidence intervals (CI) and p-values of the likelihood ratio test are given. All analyses were performed on SPSS system, version 20.

### Prognostic score for survival after reirradiation

To estimate patients’ prognosis a sum of the following values is calculated: for histology, glioblastoma is rated as 2 and WHO grade III tumors as 1. With respect to age, patients younger than 50 are given 0 points and age 50 or older is scored with 1. Time between initial radiotherapy and reirradiation is counted as 0 if 12 or more months and as 1 if the time interval is less than 12 months. A summative value of 1 represents favorable, of two intermediate and of 3/4 poor prognosis [3].

## Results

### Outcome

**Figure 1:**
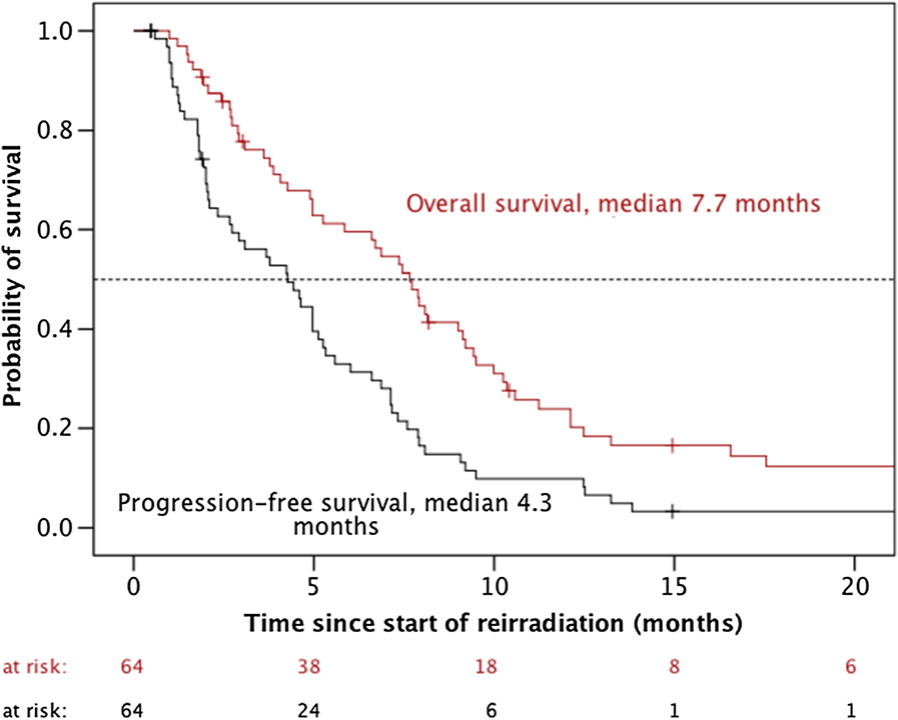
Progression-free and overall survival of 64 patients treated for recurrent high-grade gliomas using a second series of fractionated external beam radiotherapy with or without concurrent/subsequent chemotherapy.

At a median follow-up time of 7.5 months across all patients (range: 1.0 – 51.4 months), 60/64 patients (93.8%) experienced a progression and 55/64 patients (85.9%) died. Median PFS was 4.3 ± 0.9 months. PFS rates at 6 and 12 months were 33 ± 6% and 10 ± 4%, respectively. Median OS was 7.7 ± 0.7 months. OS rates at 6 and 12 months were 60 ± 6% and 24 ± 6%, respectively (Figure 1).

### Prognostic factors for OS in univariate analysis

#### Categorical variables, Kaplan Meier method, log rank test

**Figure 2:**
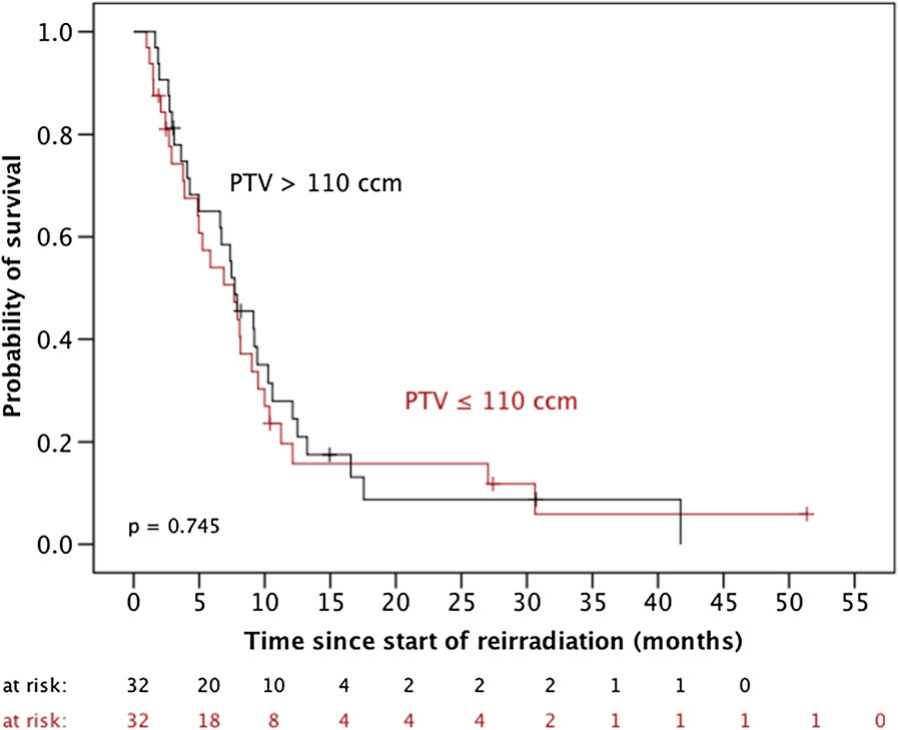
Influence of the PTV size on overall survival after reirradiation.

**Figure 3:**
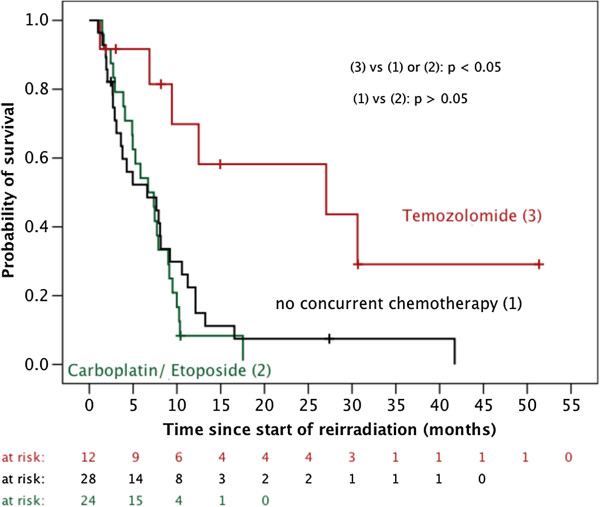
Influence of concurrent chemotherapy on overall survival after reirradiation.

Neither gender (p = 0.669) nor concurrent chemotherapy with carboplatin/etoposide (p = 0.294), the PTV size (cut-off 110 ccm) (Figure 2) or a long period of time between the end of initial radiotherapy and reirradiation (cut-off 1 year) (p = 0.140) did impact OS. In contrast, univariable survival analyses identified younger age (cut-off 50 years) (p = 0.015), WHO grade III histology (p = 0.009), favorable Karnofsky performance score (cut-off 70%) (p = 0.002), complete resection prior to reirradiation (p = 0.034) and concurrent chemotherapy with temozolomide (vs. no concurrent chemotherapy: p < 0.006 and vs. concurrent chemotherapy with carboplatin/etoposide: p = 0.001) as positive prognostic markers (Figure 3 and Table 2).

### Continous variables, Cox proportional hazards regression analysis

On univariate cox regression analyses age (p = 0.005, hazard ratio = 1.03 per year, 95% CI: 1.01-1.05) and the Karnofsky performance score (KPS) (p = 0.001, hazard ratio = 0.75 per 10 points, 95% CI: 0.64-0.89) influenced OS, whereas the size of the PTV did not (p = 0.607). One year of age increased the risk of death hence by 3% and 10 years of age by 34% (1.03^10^ = 1.34). Accordingly, the risk of death was reduced by 25% if the KPS increased by 10 percent (0.97^10^ = 0.74).

### Prognostic factors for overall survival in multivariate (cox proportional hazards regression) analysis

On multivariate analysis, female gender, young age, WHO grade III histology, favorable Karnofsky performance score and complete resection prior to reirradiation were identified as positive prognostic markers. In contrast concurrent chemotherapy, the time interval between first-line and salvage radiotherapy and the size of the PTV did not influence outcome.

### Reassessment of the prognostic score for overall survival after reirradiation of relapsed gliomas

**Figure 4:**
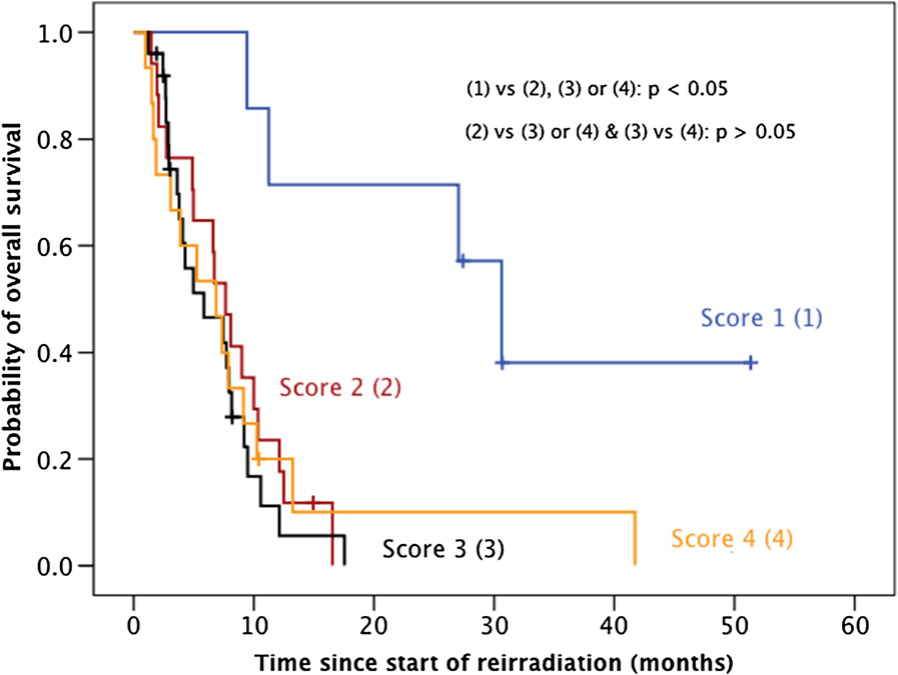
Re-assessment of the prognostic score recently suggested by Combs et al. to predict overall-survival after reirradiation of relapsed HGG.

We furthermore tried and validated the prognostic score recently generated by Combs et al. [3] to estimate overall survival after reirradiation of recurrent gliomas. For this purpose, we first subdivided our patient cohort into four different prognostic groups. Then, by means of the Kaplan-Meier method and the log rank test, we compared the respective survival curves with each other. We did not observe significant differences (p < 0.05) in overall survival between all prognostic groups. Only patients in the most favorable prognostic group (1) (young age + favorable WHO grading + good general condition) did significantly better (Figure 4).

### Toxicity

All patients completed radiotherapy, which, in general, was tolerated well. No major acute toxicities were observed (grad III/IV toxicities according to common toxicity criteria for adverse events CTCAE v3.0) (Table 3). We did not observe any case of radiation necrosis, however, posttherapeutic MRI controls were not performed routinely, hence clinically asymptomatic lesions could not be excluded. One patient died during the third cycle of maintenance chemotherapy consisting of carboplatin/etoposide due to severe hematologic aplasia.

**Table 3:** Acute toxicity during radio (chemo)therapy (all patients, patients with PTV ≤ 110 ml and patients with PTV > 110 ml)

	**All patients (n = 64)**	**PTV ≤ 110 ml (n = 32)**	**PTV > 110 ml (n = 32)**
Headache (≤ II° CTC)	7/64 (10.9%)	2/32 (6.3%)	5/32 (15.6%)
Nausea/vomiting (≤ II° CTC)	4/64 (6.3%)	3/32 (9.3%)	1/32 (3.1%)
Seizures (≤ II° CTC)	5/64 (7.8%)	2/32 (6.2%)	3/32 (9.3%)
Skin reaction (≤ II° CTC)	7/64 (10.9%)	3/32 (9.3%)	4/32 (12.5%)
Mucosa reaction (≤ II° CTC)	2/64 (3.1%)	1/32 (3.1%)	1/32 (3.1%)
Ear toxicity (≤ II° CTC)	0/64 (0%)	0/64 (0%)	0/64 (0%)
Infections (≤ II° CTC)	3/64 (4.6%)	1/32 (3.1%)	2/32 (6.3%)

*PTV* planned target volume, *CTC* common toxicity criteria version 3.0.

## Discussion

### General aspects

As stated above, reirradiation has been widely accepted as useful therapeutic option in the salvage treatment of progressive high-grade gliomas. The National Comprehensive Cancer Network (NCCN) guidelines for recurrent or progressive glioblastoma multiforme recommend to “consider”a second course of radiotherapy in local recurrence, especially if there was a long interval since prior irradiation and/or if there was a good response to first-line treatment [18]. A recent review concluded that patients with a KPS greater than 60%, progression more than 6 months from time of surgery and a tumor size of up to 40 mm were the best candidates [12]. Median KPS in our cohort was 90% (range 40 – 100%) and median time interval between initial surgery and first progression 13 months (range, 2 – 145 months). However, presuming spherical tumor shape and a safety margin of 1.0 cm from GTV to PTV, at least the latter criterion may not have been fulfilled in approximately half of our patients. This is also reflected by the fact that most of our patients (91%) underwent a second course of conventional 3D-conformal radiotherapy instead of stereotactic treatment, which is usually applied in smaller tumors (≤ 4 cm).

### Outcome in terms of overall survival and toxicity

The overall survival after reirradiation in our cohort was relatively poor but comparable to previous studies. Focussing on our glioblastoma patients only, overall survival at 12 months was 19 ± 6%. In contrast, a recent review summarizing the data of 14 reirradiation studies with a total of 300 glioblastoma patients reported survival rates at 12 months ranging between 18 and 48%. However, most of these studies only included highly selected patients with small tumor volumes suitable for stereotactic radiotherapy, brachytherapy or radiosurgery and patient numbers were small [19].

The acute radiation-induced toxicity in our cohort during and shortly after the second course of radiation therapy was negligibly low (Table 3). Moreover, to the best of our knowledge, radiation necrosis did not occur in any patient in the further course of disease. This fits well with the conclusions drawn by two important reviews on normal brain tissue tolerance which stated that a 10% incidence of radiation-induced necrosis has to be expected at a cumulative normalized total dose (fraction dose, 2 Gy; α/β, 3 Gy) of 90 Gy, but just 1–2 years after irradiation, i.e., in progressive high-grade glioma patients after the expected survival time. Unfortunately, the impact of the planning target volume (PTV) on the risk for necrosis was not elaborately discussed [16,20].

### Role of concurrent chemotherapy

In our cohort concurrent chemotherapy with temozolomide was associated with longer OS in uni- but not in multivariable analysis. Concurrent chemotherapy with carboplatin/etoposide did not influence OS. This may reflect that young patients in good general condition, i.e. with a more favorable prognosis, tended to receive outpatient radiotherapy with concurrent oral temozolomide whereas older patients in worse general condition tended to be hospitalized for simultaneous radiochemotherapy with carboplatin/etoposide or for reirradiation only. Although numerous studies on concurrent radiochemotherapy as salvage treatment in relapsed high-grade gliomas have been published so far, there is at best little evidence for a favorable impact on OS. We identified four studies evaluating retrospectively the potential benefit of concurrent/subsequent chemotherapy by comparison of survival times (Additional file 2: Table S2). In the analysis performed by Ernst-Stecken et al. chemotherapy did not influence survival. However, the number of patients was limited (n = 15) and the statistical analysis depicts methodological weaknesses, as survival times were compared by the Kaplan Meier method and log rank test instead of time-dependent cox regression [11,21]. Fokas et al. treated 53 patients with recurrent glioblastoma multiforme by hypofractionated stereotactic radiotherapy. Twenty-five patients received different chemotherapy regimens at the time of reirradiation (8× temozolomide, 9× nimustine & teniposide (ACNU/VM 26), 5× procarbazine & vincristine (PCV), 3× sequential treatment with temozolomide/nitroseureas). Addition of chemotherapy did not affect survival (p = 0.1466) [7]. A similar analysis was done by Fogh et al. who reirradiated 147 recurrent high-grade gliomas. 48 patients received different regimes of concomitant chemotherapy. Cox regression models were used to analyze survival outcomes. There was no significant benefit of chemotherapy in this population when analysis was controlled for other prognostic factors [6]. Grosu et al. reported a series of 44 patients, in which 29 patients (66%) had received one to two cycles temozolomide before and four to five cycles after reirradiation. In contrast to the previously mentioned studies, temozolomide was associated with better survival in the uni- and multivariate model [8].

### Prognostic factors

Our analysis demonstrated, that there is a variety of factors that have prognostic relevance for overall survival after salvage treatment. These factors are discussed in the following section.

### Target volume

We identified seven studies assessing the influence of the size of the target volumes on survival. Results were partly conflicting. Approximately 500 patients had been taken into account in total. Only one study reported a significant correlation between smaller gross/planned target volumes and better survival on multivariate analysis [6]. Two other studies found a tendency towards better survival in patients with smaller PTVs on univariate analysis [9,10]. According to the remaining four analyses the size of the target volumes (1× GTV, 3× PTV) was not associated with survival differences [4,7,8,22]. In our cohort the size of the PTV did not influence survival. Apart from Henke et al. and Bartsch et al. the previously mentioned groups used stereotactic radiotherapy techniques and irradiated much smaller PTVs (range of median PTVs 15 ml [10] – 50 ml [23]) than we did. We used stereotactic techniques only in exceptional cases. Median PTV was 110.4 ml. Nevertheless we did not observe major toxicities. Moreover median OS in our patient cohort (7.7 months) seems comparable to the OS observed in other series (range of median OS 7 months [22] – 16 months (only WHO grade III) [5]) taking into account the favorable influence of lower WHO grading.

### Gender

Most previous studies did not assess the possible influence of gender on OS after reirradiation of high-grade gliomas. We identified only four analyses evaluating that issue [23-26]. None of them found an impact of gender, however, in all cases only univariable analysis had been performed. In contrast to our preliminary findings in univariate analysis which were in line with the above mentioned results, multivariate (cox proportional hazards regression) analysis surprisingly revealed a positive impact of female gender on OS in our cohort. Without doubt our findings need to be reassessed by other working groups.

### Age and Karnofsky performance score

In our study young age and a favorable Karnofsky performance score positively influenced OS in both uni- and multivariable analysis. These findings are in agreement with the literature [1,6,7,24,27]. Nevertheless, at least in multivariate analysis, some authors could not confirm the positve prognostic value of young age and a favorable KPS [8,9].

### WHO grading at latest available histology

In our cohort WHO grade III tumors were associated with better OS after reirradiation on uni- and multivariable analyses. However, the results of previous studies concerning the favorable impact of WHO III grading are conflicting. Some authors confirm our results [1,10,24,26-28] whereas others do not [6,8,11,19,23,25]. One study reported that median survival time from the start of reirradiation was 10 months for patients with grade III tumors and 11 months in grade IV patients. However, the difference did not reach statistical significance [6].

### Time intervall between initial radiotherapy and reirradiation

In our cohort the time intervaI between initial radiotherapy and reirradiation did not impact OS on uni- and multivariate analysis. These findings are in line with the results of six previous studies [3,9,10,24,27,29], at least on multivariate analysis. Interestingly all authors chose a relatively short time interval (range 10 – 20 months) as cut-off for statistical assessment. In the analysis of Fogh et al. a time interval < 6 months was correlated with better survival on multivariate analysis [6]. In contrast in two series a longer time interval was associated with better survival on both uni- and multivariable analysis. However, here the authors chose relatively long time intervals as cut-off for statistical assessment (range 24 months – 36 months) [8,19].

### Extent of salvage surgery

The role of salvage surgery in recurrent or progressive high-grade glioma is controversially discussed. Some authors point out that the potential benefit achieved by tumor resection may be limited by perioperative morbidity and mortality [5,9,30-32]. In contrast, Bartsch et al. observed an improvement of survival if surgery was a component of salvage treatment [22]. In our cohort fourty-three patients underwent surgery before reirradiation. Survival was not improved (p = 0.479). This is in line with data of some other groups [7,10,29]. However, upon closer examination the extent of surgery affected outcome. Patients with complete resection depicted a median OS of 17.5 months whereas in patients with incomplete or without resection median survival was only 7.4 months (p = 0.034). In summary, our data suggests that second surgery is of limited use if complete resection cannot be achieved.

### Prognostic score

The most convincing way to demonstrate the success of a possible prognostic score is to prove its ability to discriminate different prognostic groups in a patient cohort which is independent from its development [33,34]. However, solely for the generation of a prognostic score, a very large number of patients is needed. Simon and Altman recommended at least ten times the number of events (e.g. deaths) in comparison to the number of potential prognostic factors as a “reasonable standard” [35]. Reirradiation of high-grade gliomas is rarely performed. Thus, it makes sense that several major centers are involved in the generation and validation of a scoring system to predict the benefit of reirradiation in high-grade gliomas. Combs et al. encouraged us kindly to validate the recently presented Heidelberg prognostic score [3] with our independent cohort. However, the subclassification of our patients into the four scoring groups did not demonstrate a convincing correlation with overall survival after reirradiation in visual validation and in log rank test (Figure 4). This is most likely due to the small amount of patients included into the analysis, as well the fact that only patients with high-grade histology were included. It is known that histology plays a major prognostic role, and the score was established in a patient cohort with different histologies. Since the focus of the present analysis is the evaluation of the outcome of patients with a high-grade gliomas, the score might not help to distinguish between this subgroups of patients, underlining the importance of histological classification.

## Conclusions

Outcome of our patients was comparable to survival rates reported in previous studies. Even in case of large target volumes (PTV > 110 ml, Table 3) reirradiation seems to be feasible. In the setting of reirradiation the benefit of concurrent chemotherapy needs to be determined. A reliable prognostic score might be the most robust way for prognostication. We validated a recently established prognostic score [3] with our independent but relatively small patient cohort. Our preliminary findings suggest that its ability to discriminate between different prognostic groups appears to be limited in our cohort. However, a reassessement in a much larger cohort seems to be necessary.

### Ethical statements

Written informed consent was obtained from all patient for scientific evaluation of their data. This work is in compliance with the principles of the Declaration of Helsinki and its later amendments.

## Competing interests

The authors declare that they have no competing interests.

## Authors’ contributions

FS performed the retrospective chart review and drafted the manuscript. IZ was in charge of the statistical evaluation. KM was responsible for the conception of the study and the statistical evaluation and drafted the manuscript. Manuscript editing: AS and AvB. All authors read and approved the final manuscript.

## Supplementary Material

Additional file 1: Table S1 Studies evaluating the influence of the size of the planned target volume (PTV) on overall survival after reirradiation of relapsed HGG. Click here for file

Additional file 2: Table S2 Studies evaluating the influence of concurrent chemotherapy on overall survival after reirradiation of relapsed HGG. Click here for file
