# Supplementary material for: Reirradiation in progressive high-grade gliomas: outcome, role of concurrent chemotherapy, prognostic factors and validation of a new prognostic score with an independent patient cohort
Source: Radiat Oncol. 2013 Jul 3;8:161. doi: 10.1186/1748-717X-8-161 (PMC3707836; doi:10.1186/1748-717X-8-161)
Supplement: Additional file 1: Table S1 — Studies evaluating the influence of the size of the planned target volume (PTV) on overall survival after reirradiation of relapsed HGG. [file 1748-717X-8-161-S1.docx]

| **author** | **n = (total/small/large)** | **median PTV (ml)** | **cutoff (ml)** | **univariate** | **multivariate** |
| --- | --- | --- | --- | --- | --- |
| Combs [[9](#_ENREF_9)] | 172**/-/- | 49 (range 2.5-636) | none | **no** (p = 0.497) | not done |
| Fokas [[13](#_ENREF_13)] | 53/26*/27* | 35 (range 3-204) | 30* | **no** (p = 0.469) | not done |
| Grosu [[16](#_ENREF_16)] | 44/-/- | GTV: 15 (range 1 – 61) | none | **no** (GTV !) | not done |
| Henke [[18](#_ENREF_18)] | 31/-/- | 55 (0.9 – 277) | 30  continous | **yes** (p = 0.01)  **no** (p = 0.08) | not done  not done |
| Vordermark [[33](#_ENREF_33)] | 19/9/10 | 15 (range 4 – 70) | 15 | **no** (p = 0.59) | not done |
| Fogh [[12](#_ENREF_12)] | 147/-/- | 22 (range 0.6 – 104) | none | not done | **yes** (p = 0.025) |
| present study | 64/32/32 | 110 (range 1.8 – 378) | continous | **no** (p = 0.607) | **no** (p =0.745) |

*tumor volume, ** 71 grade II tumors included

PTV = planned target volume

GTV = gross tumor volume
