# Supplementary material for: Reirradiation in progressive high-grade gliomas: outcome, role of concurrent chemotherapy, prognostic factors and validation of a new prognostic score with an independent patient cohort
Source: Radiat Oncol. 2013 Jul 3;8:161. doi: 10.1186/1748-717X-8-161 (PMC3707836; doi:10.1186/1748-717X-8-161)
Supplement: Additional file 2: Table S2 — Studies evaluating the influence of concurrent chemotherapy on overall survival after reirradiation of relapsed HGG. [file 1748-717X-8-161-S2.docx]

| **author** | **n = (total/chemotherapy/**  **no chemotherapy)** | **chemotherapy**  **regimen** | **univariate** | **multivariate** |
| --- | --- | --- | --- | --- |
| Ernst-Stecken [[11](#_ENREF_11)] | 15/8/9 | various regimes | **no** (p=?) | not done |
| Fogh [[12](#_ENREF_12)] | 147/48/99 | various regimens | not done | **no** (p = 0.791 |
| Fokas [[13](#_ENREF_13)] | 53/25/28 | various regimens | **no** (p = 0.1466) | not done |
| Grosu [[16](#_ENREF_16)] | 44/29/15 | TMZ | **yes** | **yes** (p = 0.04) |
| present study | 64/36/28 | TMZ | **yes** (p=0.006) | **no** |

TMZ = temozolomide
